# A New Productive Approach and Formulative Optimization for Curcumin Nanoliposomal Delivery Systems

**DOI:** 10.3390/pharmaceutics15030959

**Published:** 2023-03-16

**Authors:** Raffaella De Piano, Diego Caccavo, Gaetano Lamberti, Katrien Remaut, Hanne Seynaeve, Anna Angela Barba

**Affiliations:** 1 Dipartimento di Ingegneria Industriale, Università degli Studi di Salerno, Via Giovanni Paolo II n.132, 84084 Fisciano, Italy; 2 Eng4Life srl, University Spin-Off, Via Circumvallazione n.39, 83100 Avellino, Italy; 3 EST srl, University Spin-Off, Via Circumvallazione n.39, 83100 Avellino, Italy; 4 Department of Pharmaceutics, Ghent University, Ottergemsesteenweg 460, 9000 Gent, Belgium; 5 Dipartimento di Farmacia, Università degli Studi di Salerno, Via Giovanni Paolo II n.132, 84084 Fisciano, Italy

**Keywords:** nanoliposomes, curcumin, nanotechnologies, simil-microfluidic technology, antioxidant activity, anticancer, nutraceuticals

## Abstract

The use of natural resources and the enhancing of technologies are outlining the strategies of modern scientific-technological research for sustainable health products manufacturing. In this context, the novel simil-microfluidic technology, a mild production methodology, is exploited to produce liposomal curcumin as potential powerful dosage system for cancer therapies and for nutraceutical purposes. Through simil-microfluidic technology, based on interdiffusion phenomena of a lipid-ethanol phase in an aqueous flow, massive productions of liposomes at nanometric scale can be obtained. In this work, studies on liposomal production with useful curcumin loads were performed. In particular, process issues (curcumin aggregations) were elucidated and formulation optimization for curcumin load was performed. The main achieved result has been the definition of operative conditions for nanoliposomal curcumin production with interesting loads and encapsulation efficiencies.

## 1. Introduction

In recent years, the demand of dietary supplements such as phytomedicines and vegetal ingredients from nutraceutical industries has grown enormously. This depends upon the rising worldwide community request of natural, safe, and healthy bioactive products. The global nutraceutical market of botanical drugs, based on traditional herbal medicine, is undergoing a huge expansion hand in hand with the development of new technologies. The final goal is focused on the realization of more highly bioavailable dosage forms of nutrients and therapeutics that are able to improve people health, longevity, and quality of life [1,2,3,4]. Thus, this dual aspect, namely the use of natural resources for healthy benefits and updating of technologies, is influencing the strategies of modern scientific-technological research. Research responds to the goal of the sustainable development objectives at the center of the current socio-economic policy promoted by the United Nations as a strategy “to achieve a better and more sustainable future for all” [5].

**Figure 1:**
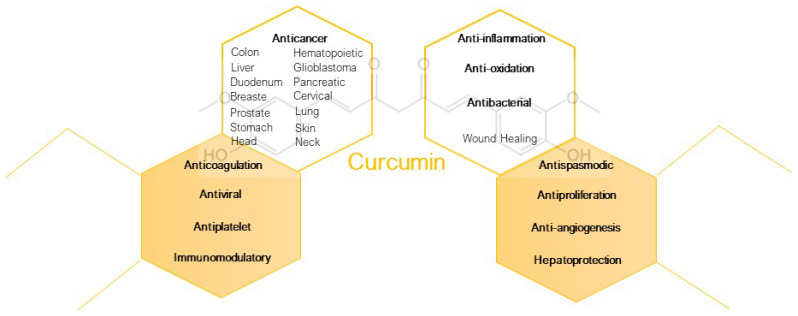
Pharmacology benefits of curcumin [8,9,10].

In this context, natural resources, due to their variety and peculiar richness in secondary metabolites, are currently objects of scientific and industrial interest. Among the countless vegetal species with formidable biological features, curcumin (CUR), the main components of curcuminoids, polyphenolic compounds found in the rhizomes of turmeric (Curcuma longa, Zingiberaceae family), is one of the natural compounds that have been extensively studied from a pharmacological perspective, and is well known as a spice and as a dye [6,7]. In the latest scientific literature, more than three thousand papers (source: WOS core collection, curcumin as keyword, years 2022–2023) testify the great interest in this natural compound. The reasons for this considerable attention are the ascertained therapeutic efficacy in arthritis, liver, and neurodegenerative diseases; in several types of cancers, obesity, wound-healing, anti-inflammatory treatments; and as an antibacterial, antioxidant, antispasmodic, and anticoagulant agent [8,9,10]. In addition, recently, studies on CUR’s preventive effects on extensive immunosuppression are ongoing to investigate therapeutic potential against SARS-CoV-2 (COVID-19) [6]. In Figure 1, the pharmacology benefits of curcumin are summarized.

Despite its beneficial properties, CUR uses in clinical applications are strongly limited due its high hydrophobicity (0.125 mg/L, [9]). This poor solubility in aqueous media results in low bioavailability into plasma and living target tissues. Curcumin also has rapid metabolism, which significantly affects its half-life and bioavailability. Moreover, as for most the antioxidant agents, curcumin is unstable and quickly degraded through oxidative processes [7] during manufacturing and storage [9]. In this regard, to overcome curcumin dosage limitations, evolutions of nanotechnologies and of their outputs, i.e., nanoparticles (NPs), are extensively discussed in the literature in terms of possible benefits and disadvantages [10,11]. Nanoparticles offer considerable advantages over conventional drug-delivery systems due to their small size and consequently large surface area. They can allow transportation and release of drugs with tailorable release kinetics of active ingredients to target side as well as enhanced solubility and stability of most drugs, resulting in improved pharmacokinetic profiles [12,13]. In particular, dedicated investigations for curcumin delivery have emphasized that nanoliposomes are the better administration platforms, even if their use can also manifest adverse effects [9,10,11,14]. Liposomes are artificial self-assembled colloidal particles consisting of one or more lipid bilayers surrounding an aqueous core. They are useful as biocompatible drug-delivery vehicles for hydrophilic, amphiphilic, and lipophilic molecules which are poorly absorbed and/or rapidly metabolized in their naked form (such curcumin). Due to their low intrinsic toxicity and immunogenicity and their resemblance to cell membranes in terms of structure and composition, a characteristic that favors the drug penetration through biological barriers, liposomes are attractive candidates in the controlled release of many kinds of active ingredients [15,16,17]. Moreover, liposomes’ size, which has a key role in carriers’ uptake after their administration, can be easily modified according to need, i.e., it has flexible features. In particular, liposomes of nanometric size are preferred. As discussed above, nano dimensions allow larger interfacial surface area that improves solubility, bioavailability, and release properties [18]. Nanoliposomes are able to maintain the transparency of clear beverages in the case of nutrients enrichment [19] and also enhance the intestinal absorption of the active principles [20]. It is noteworthy to mention that nanoliposomal formulations have successfully been used in clinical applications as an FDA-approved nanocarrier for a variety of drugs [13]. Lipocurc^TM^ is the most known curcumin liposomal product that has undergone clinical trials [10,14]. To cite several outcomes, nanoliposome-based delivery systems for CUR have revealed: enhanced bioavailability, better anticancer effects [9,11]; lower adverse effects but also higher cytotoxicity (trials on cell lines for several cancer type) [9]; enhanced anti-inflammatory proprieties (test in vivo) [21]; better internalization by cells into cytoplasm, and attenuation of neuroinflammatory reactions (in cell lines/in murine brain tissue) [13]. 

Since the introduction of liposomes to the scientific community, many techniques and methodologies have evolved for their production such as the Thin Film Hydration (TFH) or Bangham method [22], the Ethanol Injection (EI) [23], the High-Pressure Homogenization (HPH) [24], the Reverse Phase Evaporation (RFE) [25], the Supercritical Fluid (SF) [26], the Microfluidic Channel (MC) [27], just to cite a few. Details and discussions on principle and equipment of the reported production methods can be found in literature sources as books and reviews [28,29,30]. Despite the ever-increasing interest in the field and the enormous research that has been undertaken, the manufacturing process represents the principal barrier in the large scale production of liposomes. Ultimately, liposomes production methods are bulk discontinuous techniques characterized by long times of process, high-energy request and the use of toxic solvents or low/high values of pH and pressure during the preparation which affect the chemical structure of the entrapped substance [18,31]. Moreover, the majority of these methods suffer from the impossibility to scale up the process, and are characterized by small volumes in output whereby they cannot give products directly at nanoscale, thus requiring further laborious steps for vesicles sizing, i.e., sonication or membrane extrusion. Even the most recent techniques, i.e., based on microfluidics approach, require very expensive devices which are difficult to scale up.

Recently, considering the lack of a continuous and practical large-scale manufacturing technique, a new simil-microfluidic method, characterized by potential continuous regime, massive and rapid production features, was developed. It was described in detail in [32] and in the patent document WO2019049186 [33]. The developed method exceeds the limits of conventional techniques also offering lower production costs and environmental impact by avoiding the use of special micro-fabricated devices and drastic process conditions. 

This study is focused on the production aspects of liposomal curcumin in aqueous prevalent medium. It presents, in particular, the application of the novel simil-microfluidic method, as mild and throughput technology, to produce encapsulated curcumin in nanoliposomal structures. After a process-issues presentation, with the aim to optimize the load of curcumin component and its encapsulating capability, several investigations varying curcumin concentration were performed. All types of achieved products were characterized in terms of size, polydispersity index, load, and encapsulation efficiency. 

## 2. Materials and Methods

### 2.1. Materials

Cholesterol (CHOL) (CAS no. 57-88-5), L-a-Phosphatidylcholine (PC) from soybean, type II-S, 14–23% choline basis (CAS no. 8002-43-5), ethanol of analytical grade (CAS no. 64-17-5) and curcumin (CUR) (CAS no. C1386) were acquired from Sigma Aldrich (Milan, Italy) and used as received. Deionized water was product by lab deionizer. 

**Figure 2:**
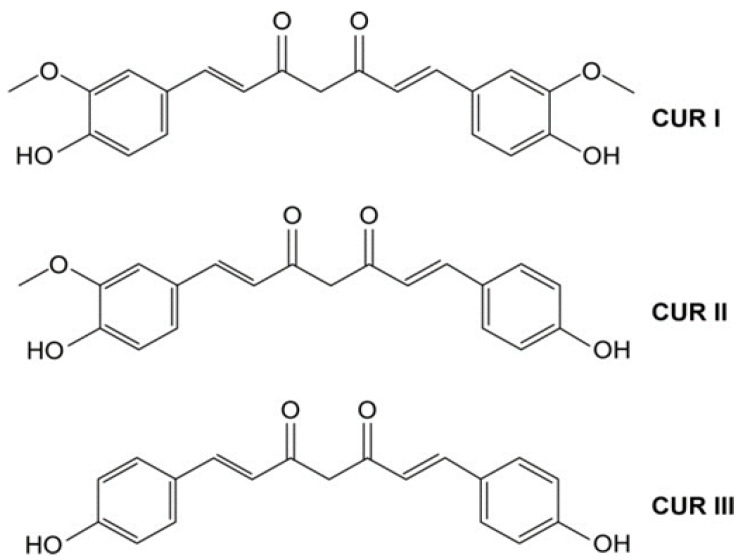
Curcumin structure: curcumin (CUR I), demethoxycurcumin (CUR II) and bisdemethoxycurcumin (CUR III).

Curcumin, also called diferuloylmethane, is a yellow polyphenolic powder extracted from the rhizome of *Curcuma longa* and other Curcuma species [9,34]. Three are the main types of curcuminoids: curcumin (CUR I), desmethoxycurcumin (CUR II), and bisdemethoxycurcumin (CUR III) (structures in Figure 2). Their abundance in turmeric is around 77, 17, and 3% respectively [9]. The most abundant one is thus CUR I, a lipophilic molecule with a molecular weight of 368.38 g/mol and a molecular formula of C_21_H_20_O_6_ [35]. The curcumin (CUR I) structure is a linear diarylheptanoid which consists of two aromatic rings (aryl groups) joined by a seven carbon-unit chain (3) with hydroxyl and carbonyl functional groups as shown in Figure 2 [36]. In Table 1, the main properties of curcumin are summarized [37].

### 2.2. Production Method 

#### 2.2.1. Liposomes Production by Simil-Microfluidic Technology: Principles and Setup

**Figure 3:**
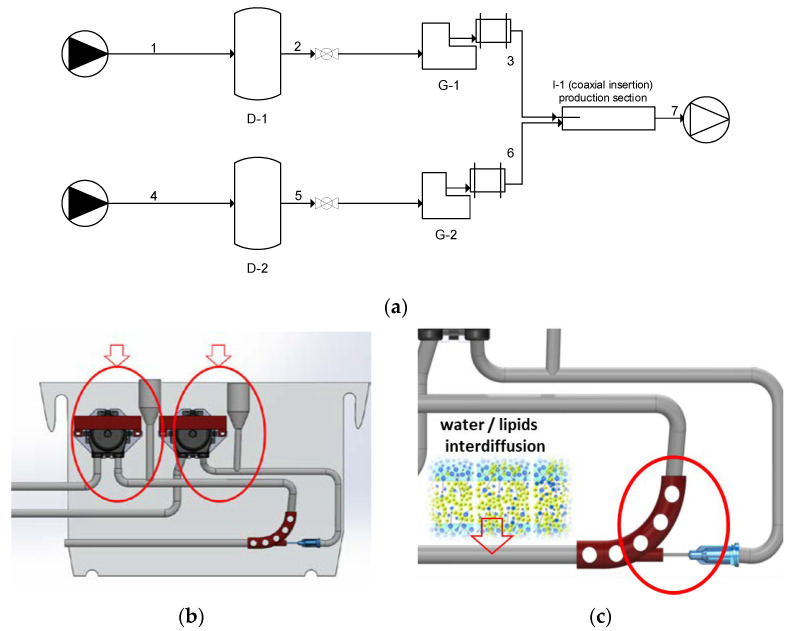
(**a**) Simil-microfluidic piping representation (adapted from [33])**:** Section of nanoliposome preparation: (1–2–3) lipids/ethanol feed line; (4–5–6) water feed line; (D-1 and D-2) feed tanks; (G-1 and G-2) peristaltic pump-dampener groups; (I-1) injector (production section); (7) water/ethanol nanoliposome suspension; (**b**) Inlets flows. (**c**) Close-up of injection point: coaxial flows insertion.

To address the limitations of existing techniques for producing nanoliposomes—such as the need for harsh operating conditions, toxic solvents, multiple post-processing steps, poorly controlled conditions, low output volumes, and high costs associated with microfluidic devices—a new technology called “simil-microfluidic” has been developed [16,32,38]. This novel technology allows for the production of homogeneous, antioxidant-loaded nanoliposomes in a single step at room temperature, directly at the nanoscale. The process involves the controlled contact of two phases through a coaxial insertion system using pumps to introduce the lipid and hydration solution feeds. A diagram of the setup piping is shown in Figure 3a, and details on the inlets systems (pump-dampener groups) and lipid flow insertion are shown in Figure 3b,c, respectively. The simil-microfluidic technology offers precise control of fluid dynamics and eliminates the need for toxic solvents, making it a promising approach for nanoliposome manufacturing.

#### 2.2.2. Massive Production 

The simil-microfluidic setup has the potential to operate continuously at high throughput by maintaining a fixed ratio between the volumetric flow rates of the two phases. In this study, batches of curcumin-loaded liposomes were produced using a hydration solution volumetric flow rate (Vhs) and lipid solution volumetric flow rate (Vls) ratio of 10:1 (Vhs/Vls). Specifically, the volumetric flow rates of Vhs and Vls were set at 45 mL/min and 4.5 mL/min, respectively. Under these conditions, a significant amount of liposomal suspensions—approximately 3 L/h—can be produced in a short amount of time.

In this study, several production batches were prepared. The first batch was produced using a recipe outlined in a previous study [16]. To prepare the lipid ethanol solution, 940 mg of PC, 188 mg of cholesterol, and 140 mg of curcumin were dissolved in 20 mL of ethanol, resulting in a theoretical concentration in CUR of 6.99 mg/mL. The hydration solution consisted of 200 mL of deionized water. The ethanol solution was stirred for an entire night to ensure complete lipid dispersion into the alcoholic phase, resulting in an initial lipid/ethanol solution concentration of approximately 50 mg/mL lipids (5 mg/mL lipids in the final hydroalcoholic solution).

Other production batches (referred to as “Prod.” in the following) of curcumin in lipids/ethanol phase were prepared with varying compositions, as shown in Table 2. The same recipe for lipid composition (PC, CHOL) was used for all production batches, but the amount of curcumin was reduced for each batch, resulting in theoretical concentrations of 4.98 mg/mL, 2.85 mg/mL, 1.42 mg/mL, 0.73 mg/mL, and 0.49 mg/mL in ethanol prior to encapsulation.

The final production batch (batch 7) was carried out to study the effect of lipid amount on encapsulation efficiency. To this purpose, a different recipe was employed. As indicated in Table 2, a theoretical curcumin concentration of 0.71 mg/mL in ethanol phase was selected (corresponding to CUR load of prod. 5), but only half of the initial amount of lipids was used.

### 2.3. Characterization Methods

#### 2.3.1. Separation Steps

Curcumin is a compound that is soluble in lipids and can be easily dissolved in organic solvents such as ethanol, methanol, and acetone. In particular, its solubility in ethanol is reported to be 10 mg/mL, as shown in Table 1. As reported in the Introduction, curcumin is insoluble in water, and thus, it tends to aggregate in hydrophilic solvents. Its aggregates have a typical sharp needle-structure [35]. This behavior in aqueous environments can cause significant issues during the manufacturing and storage stages, as well as in curcumin assay protocols, and there are gaps in the scientific literature regarding this point.

During our experimental work on nanoliposome preparation using an aqueous medium, we encountered issues with the formation of aggregates. To distinguish between aggregates and vesicular structures, a filtration stage was added to the preparative protocol. Before and after filtration, suspensions were characterized to assess the impact and effect of the separation stages.

Initially, all the liposomal suspensions produced were subjected to a rough syringe filtration using a membrane filter (Whatman^®^, Merk, Maidstone, UK) with a pore size of 450 nm. This was done to retain any coarse structures that might be present in the suspensions.

Next, to separate the pellet from the supernatant, we used an additional filtration step employing tangential flow filtration (TFF) membrane. This process produced a retentate (pellet) and a permeate (supernatant or filtrate). The TFF membrane was placed in a custom-made filtration loop that included two plungers operating in opposite directions. When one plunger fills, the other empties, creating a continuous flow. This approach is efficient and easy to use [39]. The TFF membrane (Minimate TFF Capsule with Omega membrane—modified polyether-sulfone) allows for continuous tangential flow of the liposomal suspension over the membrane. The tangential flow filtration method was preferred over the classic ultracentrifugation because it is gentler on the vesicle structure and reduces the risk of contamination between the pellet and the supernatant. It is worth noting that the centrifugation method deposits all particles on the bottom, making it impossible to discriminate between particles or different aggregates of higher dimensions, as occurs with CUR in aqueous bulk, which can falsify encapsulation yields. We opted for tangential flow filtration instead of crossflow filtration because the latter can quickly saturate the membrane and cause vesicle rupture due to increasing pressure over the membrane.

#### 2.3.2. Vesicles Size and Suspension Inspection

For the nanolipid vesicles, the Z-average size, which is defined as the average hydrodynamic diameter, and the numerical size distribution were measured using the dynamic light scattering (DLS) method. The DLS measurements were conducted under environmental conditions after dilution and sonication of both the liposomal suspensions and syringe filtrates with distilled water in ratios ranging from 1:3 to 1:4. Each sample was measured at least three times, and the results were expressed as average values.

The produced suspensions were observed by optical and transmission electron microscopy (TEM). The Leica microscope DMLP equipped with the digital camera Leica DFC 480 was used for optical microscopy investigations at a magnification of 40×. TEM images were achieved by the EM 208, Philips instrument, equipped with a camera Olympus Quemesa (EMSIS GmbH and Software RADIUS). Approximately 10 μL of washed pellet sample were diluted with distilled water and were placed on a carbon support over a copper specimen grid mesh 200 (Electron Microscopy Sciences) and, finally, negatively stained with a solution uranyl acetate (1% *w*/*v*).

#### 2.3.3. Encapsulation Efficiency and Load

Samples were taken from all liposome batches produced to measure the amount of curcumin encapsulated and unencapsulated. The sampling process included three stages:

- first, aliquots were taken from each prepared liposomal suspension batch;
- second, aliquots were taken from each syringe-filtered bulk;
- third, aliquots were taken from each TFF permeate bulk.

These samples were then subjected to spectrophotometric analysis.

Samples of the liposomal suspension were diluted in ethanol and sonicated for 1 min (at 100% amplitude -VCX 130 PB Ultrasonic Processors, 130 W, Frequency 20 kHz, Sonics & Materials Inc., Newtown, CT, USA) before undergoing UV-VIS spectrophotometric analysis (Lambda 35, PerkinElmer, Monza, Italy) to measure the encapsulated and unencapsulated amounts of curcumin. Absorption spectra were acquired between 200 nm and 600 nm, and the absorption maximal wavelength for curcumin was considered to be 426 nm, according to the literature [40].

The filtrated and permeate samples, as well as the liposomal suspension samples, were also diluted in ethanol and subjected to UV-VIS spectrophotometric analysis, with sonication applied only to the syringe-filtrated samples to disrupt the vesicles. Proper dilutions were used to stay within the calibration curve.

The percentage of drug encapsulated in liposomal vesicles, known as encapsulation efficiency (e.e.), was determined using Equation (1). This equation gives the ratio of the difference between the initial (o total drug) and the drug detected in the permeate, to the initial amount of drug added in the formulation:

(1) $$e.e.\left(\%\right)=\frac{Total,mg-Permeate,mg}{Total,mg}\cdot 100$$

Since the filtration steps can retain CUR, Equation (1) must take in account the two steps of separation. Thus, the effective efficiency has to be calculated as the product of the first efficiency, the syringe separation, and the efficiency after the TFF, by Equation (2):

(2) $$e.e.\left(\%\right)=\left[\frac{Curinsyringefiltrate,mg}{TotalCUR,mg}\right]\cdot \left[\frac{CURinsyringefiltrate,mg-CURinTFFpermeate,mg}{Curinsyringefiltrate,mg}\right]\cdot 100$$

In particular, in Equation (2) the first part pertains to the separation efficiency of the filter syringe (e.s. syringe), while the second part refers to the TFF separation efficiency (e.e. TFF). 

The term “theoretical load” denotes the initial quantity of CUR present in the formulation divided by the total mass of all the ingredients utilized:

(3) $$TheoreticalLoad\left(\%\right)=\frac{TotalCur,mg}{TotalCur,mg+lipidsPCCHOL,mg}\cdot 100$$

Effective or practical load was then determined as the effective encapsulation efficiency multiplied by the theoretical load, as reported in Equation (4):

(4) $$EffectiveLoad\left(\%\right)=e.e.\cdot TheoreticalLoad$$

Table 3 provides a summary of the composition of the seven production batches along with the corresponding theoretical load calculations.

#### 2.3.4. Stability

The features of liposomal preparations that were stored for one month after syringe-filtration were examined. The samples were kept at 4 °C and protected from light, and were visually inspected weekly to detect any potential aggregation or segregation. At the end of the fourth week, samples were collected from all the formulations and analyzed for their size and load using the same methods as for the fresh samples.

### 2.4. Statistical Analysis 

The statistical significance of the experimental data was evaluated by comparing the independent variable of the CUR/PC ratio (mg of curcumin/mg of phosphatidylcholine) with the dependent variable of the particle sizes. This analysis was performed using Student’s T-test and one-way analysis of variance (ANOVA), with statistical significance considered at *p* < 0.05.

## 3. Results and Discussion

### 3.1. Production Insights

In our simil-microfluidic apparatus, laminar flow occurs due to the small scale of the channels and low volumetric flow rates, as similarly happen into microfluidic systems, unlike the chaotic flows observed in conventional bulk preparation methods. It is noteworthy to observe that fluid dynamic in microchannels constitutes a non-trivial topic that is not referable to the classic studies of intubated flows. The definition of the fluid dynamic behavior of flows, in particular in coaxial configuration, requires attention in understanding regime developed and mixing time [41]. 

In this study, the tested operative conditions, i.e., volumetric flow rates of 45 mL/min and 4.5 mL/min Vls for aqueous (Vhs) and ethanolic phase (Vls), respectively, allow a laminar regime for the resulting hydroalcoholic suspensions (flow velocity ratio 7.56—considering, for the calculation, the mentioned rates for lipid ethanol as inner flow and water as outer flow, and the appropriate values for physical and geometric parameters for feeds and tubes; and Reynolds number 240). This result allows us to hypothesize that the mixing between the two phases is primarily governed by diffusion mechanisms. During these diffusion processes, lipid vesicles on a nanometric scale begin to form through self-assembly, according to Lasic et al.’s theory (1998) [42]. Briefly, lipids dissolved in an organic solvent are in the form of bilayer fragments (Phospholipid Bilayer Fragments, BPFs), the interdiffusion of the water and the organic solvent reduces the solubility of the lipids in the solvent and this reduction, along with the thermodynamic instability of BPFs edges, induces curvature and closure of bilayer fragments which allows the formation of liposomal vesicles.

Typically, the technique exhibits high encapsulation efficiency for lipophilic active ingredients, which are primarily dissolved in the lipid phase. However, both the molecule being encapsulated and the lipid materials’ physicochemical characteristics may impact the encapsulation yield’s success. Curcumin is a lipophilic ingredient with a quite small molecular mass: these features favor its entrapment and accommodation in phospholipidic bilayer [9,36,40]. This study aimed to determine the maximum amount of curcumin that could be added to liposomes produced by the simil-microfluidic method.

Based on the obtained results, which are discussed in detail in the following sections, the first observation was focused on the production of highly heterogeneous products. Additionally, the liposomal structures exhibited low encapsulation efficiencies and load values, which could be attributed to the formation of curcumin aggregates. To explain this, an antisolvent effect was hypothesized, in line with previous literature works on the achievable maximum load of liposomal curcumin [40,43]. When the alcoholic and water phases come into contact, curcumin and lipids gradually lose their solubility in the forming ethanol/water suspension. This effect not only promotes the closure of bilayer fragments but also leads to a “supersaturation” of curcumin, which then aggregates upon contact with the aqueous phase. These phenomena are governed by thermodynamics and kinetics, which in turn can depend on the concentration of materials (lipids, curcumin) and mixing time, respectively.

This study aimed to address the issue of curcumin aggregate formation in liposomal suspensions by introducing a separation stage to exclude large curcumin particles from the final product. This approach prevented interference with vesicle characterization protocols and misleading results on low encapsulation efficiency and load values. Then, various initial curcumin concentrations were tested to optimize the encapsulation yield limiting losses due to aggregation (as detailed in Table 3, which lists the composition of the seven production batches).

### 3.2. Liposomes Characterization

#### 3.2.1. Encapsulation Efficiency and Load

In Table 4 encapsulation efficiencies and effective loads of all production batches were reported. For the first production batch an encapsulation efficiency of 11.12% and a load of 1.23% were obtained. These values are different from those obtained by simil-microfluidic approach with other liposoluble compounds as, for example, vitamin D3, for which an efficiency of 88.4% and an effective load of 9.20% were achieved as shown in a previous work [17]. 

The nature of the molecules being encapsulated plays a crucial role in their arrangement within the liposomal bilayer. CUR, diversely from D3 that largely interacts with phospholipid bilayer [44], tends to lie perpendicular to the bilayer normal [36]. This is due to curcumin’s polar groups to be close to the hydrophilic groups of phospholipidic chains in order to form a larger number of hydrogen bonds. The described accommodation may thus limit the molecules’ inclusion.

We observed that aggregates formation is related to the amount of initial CUR. As it can be seen from the separation efficiency data (e.s.%, in Table 4), the retention of curcumin (as aggregates) decreases from batch 1 to 6, i.e., in the filtrate CUR (entrapped inside the vesicles as demonstrated by CUR absence in TFF permeate) increases its value from 11.13% to 89.74%. 

Subsequent TFF filtration allows to separate loaded vesicles from hydroalcoholic suspensions without further losses of CUR. Indeed, the detected CUR in the permeate was negligible, confirming its encapsulation in liposomal vesicles (retentate product).

In order to optimize the encapsulation efficiency of curcumin, the initial amount of curcumin was gradually decreased while keeping the masses of the other compounds fixed (as shown in Table 3 for Prod. 1–6 compositions). This approach has been adopted in research where similar liposomal formulations were used to study interaction and localization of curcumin with/in liposomal bilayers. These studies examined the effects of curcumin concentration on the average hydrodynamic diameter of vesicles [43] and their mechanical properties [40]. Curcumin has been shown to preferentially locate in the lipid bilayer of the liposome, and thus the loading capacity of the carrier is directly related to its interaction with the lipid components of the vesicles. In Karewicz et al.’s studies [43], the size of vesicles decreased with an increasing amount of curcumin, due to its condensing effect on the liposomal bilayer. Moreover, the loading capacity of curcumin in vesicles was affected by its starting amount. After reaching a critical value, higher quantities added to the formulation caused precipitation and an increase in average size, likely due to the formation of aggregates resulting from destabilization of the system. Arab-Tehrany et al. [40] observed that as curcumin was added/inserted into the lipid bilayer, both elasticity and rupture force progressively decreased. This was explained by a progressive homogenization of the lipids’ packing due to the interactions between curcumin and the alkyl chains of the lipids (hydrophobic molecules can modify the membrane curvature generating rearrangements in the packing of lipids). In addition, this effect was hypothesized to be controlled by a critical concentration of curcumin. When its amount in the membrane becomes significant, destabilizing effects occur, reducing elasticity and the resistance to rupture forces.

In this study, the effect of the curcumin starting concentration on liposomal products were elucidated by measuring loading capacity, encapsulation efficiency and particle size.

**Figure 4:**
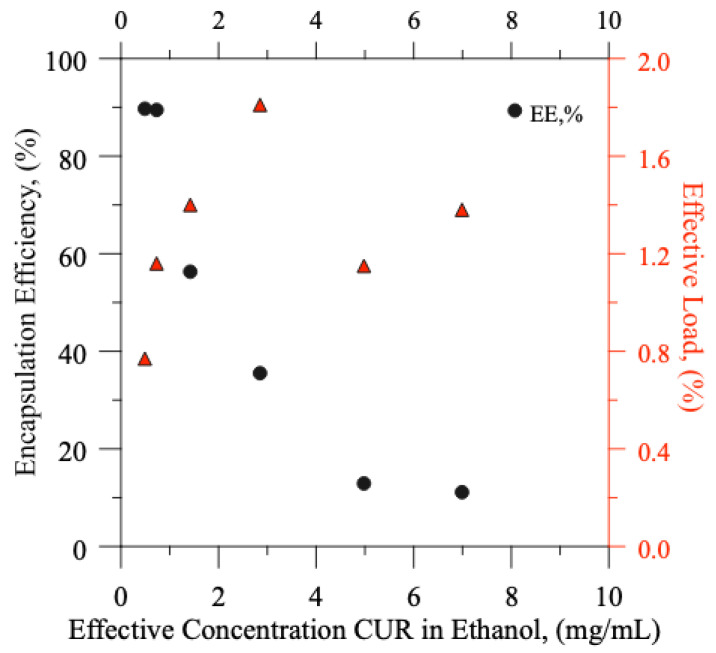
Encapsulation efficiency and effective load vs. effective curcumin concentration (in ethanol phase) of prod. batches 1–6.

In Figure 4 (and Table 4), encapsulation efficiency and effective loads vs. curcumin effective concentration were reported. An increase in encapsulation efficiency until a maximum of 89.71% (prod. batch 6) was achieved reducing the starting amount of CUR.

The load values, instead, were roughly constant with a value fluctuating around 1.3%. The achieved results confirm the presence of a “critical load-limit” with respect to the lipid composition, as shown in literature. Curcumin can be located in the bilayer until a critical amount; the highest values lead to curcumin losses for aggregations. 

The best loading capacity and encapsulation efficiency were achieved for the prod. Batch 5 (e.e. 89.47%; load of 1.15%) indicating as a load-limit, for our lipid formulation, the effective concentration of the prod. batch 4. This conclusion is also substantiated from DLS particles size analysis. 

It is worth noting that in literature articles variable efficiency of encapsulation efficiencies and loads for CUR nanovesicular systems, ranging from 54% to 95% and 1.9% to 4%, respectively, have been achieved. These remarkable outcomes were, however, obtained through the use of complex and multistep methods, involving multiple components and coating processes to ensure stability of the CUR-nanosystems [9].

#### 3.2.2. DLS Analysis and Suspension Inspections 

Table 5 reports the results of DLS investigations on size (numerical and Z-average) and PDI for particles (CUR aggregates and vesicles) suspended in the produced batches. 

**Figure 5:**
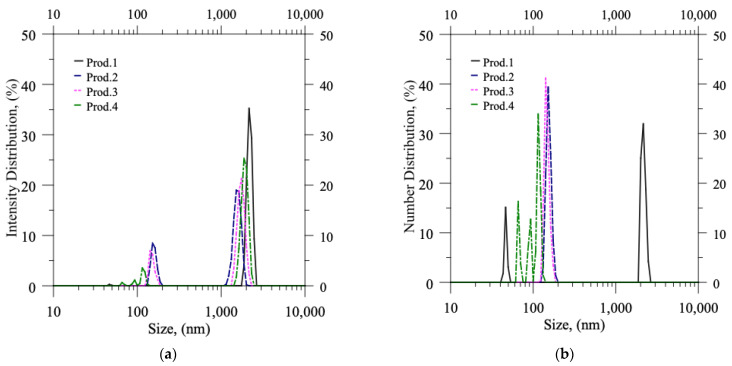
DLS measurements: probability distribution functions (PDFs) in terms of (**a**) Intensity distribution and (**b**) Number distribution versus size of particles suspended in production batches 1–2–3–4 (as produced, before filtration).

**Figure 6:**
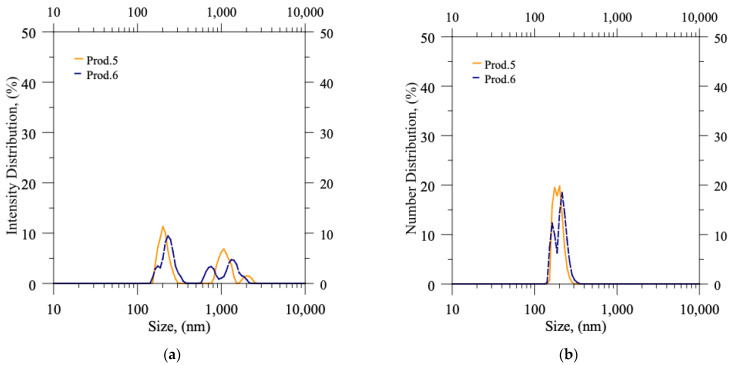
DLS measurements: probability distribution functions (PDFs) in terms of (**a**) Intensity distribution and (**b**) Number distribution versus size of particles suspended in production batches 5–6 (as produced, before filtration).

In Table 5, the column named x_1,0_ reports the mean size value of the numerical probability distribution function; the column x_mod,6_ indicates the (maximum) modal value of the intensity probability distribution function (PDF), i.e., the size corresponding to the maximum peak in the intensity PDF (q_6_: these values can be easily identified by inspection of Figure 5 and Figure 6, being the quotes of the highest peaks). It is important to note that each value (both for x_1,0_ and x_mod,6_) is the outcome of an average of three different productions that were carried out under identical operating conditions to ensure repeatability.

In DLS, the values of q_6_, which are obtained through autocorrelation function analysis, are directly proportional to the sixth power of particle size. Hence, the x_mod,6_ value, which corresponds to the modal value of the intensity probability distribution function, directly reflects the presence of large particles or aggregates. As expected, the values of x_mod,6_ decreased significantly for each experiment following syringe filtration, as seen in Figure 5 for production batches 1–4. Otherwise, in productions 5 and 6 (Figure 6), the values of x_mod,6_ were already low before the filtration step, indicating the absence of any aggregates in the produced batches.

In contrast, the values of x_1,0_, which represent the small and abundant particles, remain nearly unaffected by both the formulation parameters and the filtration process. Regardless of the production conditions, the process generates numerous small liposomes, resulting in a size mean value that generally falls between 100 and 250 nm, as indicated by the peaks located just above 100 nm in each experiment before and after filtration. The sole exception is the production batch 1, characterized by a higher concentration of CUR, in which the abundance of aggregates substantially influences the mean numerical value x_1,0_ (at least before filtration).

The stability studies performed on aged samples of all production batches (filtered samples) support the DLS data obtained for size (numerical and Z-average) and PDI of the fresh productions (filtered samples). Additionally, the CUR detected in the filtrates was negligible, indicating its stable retention within the liposomal nanovesicles under refrigerated conditions.

**Figure 7:**
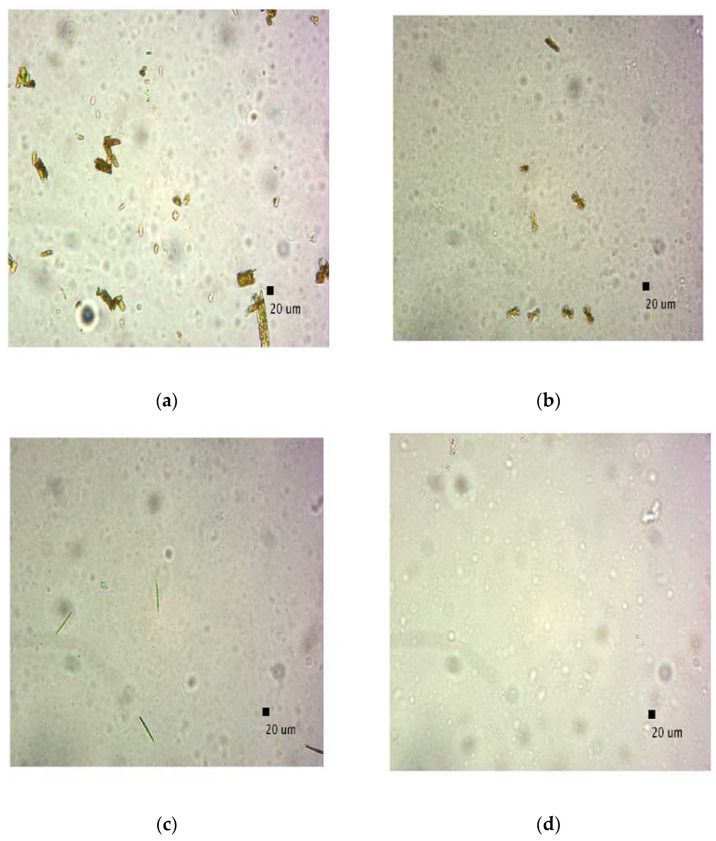
Optical microscope pictures of the different liposomial suspensions: (**a**) 6.99 mg/mL, (**b**) 2.85 mg/mL, (**c**) 1.42 mg/mL, (**d**) 0.73 mg/mL (CUR concentrations are referred to the effective values—Table 2; a dilution of 1:4 and magnification of 40× were applied).

Microscopic images were captured to validate the presence of CUR aggregates identified by DLS findings in various liposomal suspension samples (before filtration). Figure 7 displays irregular, needle-shaped aggregates in the liposomal suspensions of production batches 1 (Figure 7a), 3 (Figure 7b), and 4 (Figure 7c). The image in Figure 7d is related to the production batch 5 sample, where aggregates were not observed, as verified by DLS analysis. This finding reinforces the hypothesized critical concentration of CUR necessary for the successful implementation of the similar-microfluidic technique. In general, these results indicate the importance of optimizing the formulations of liposomal curcumin. By using appropriate concentrations, the loss of CUR as aggregates can be avoided, which are not beneficial for health purposes due their low bioavailability in “naked” form, and also reduces the impact on material costs.

**Figure 8:**
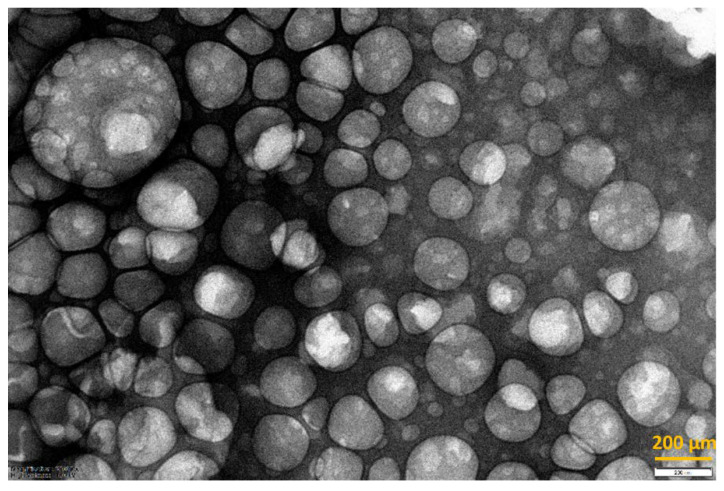
TEM picture of pellet sample from liposomial suspensions prod. batch 5.

In Figure 8 a TEM image of production batch 5 (sampling from washed pellet) is shown. The image confirms that the simil-microfluidic technique produces spherical nanosized vesicles. 

### 3.3. Effect of Lipid Concentration 

The aim of this part of the work was to assess the impact of reducing the lipid fraction (as outlined in Table 2) on the characteristics of CUR loaded vesicles. 

**Figure 9:**
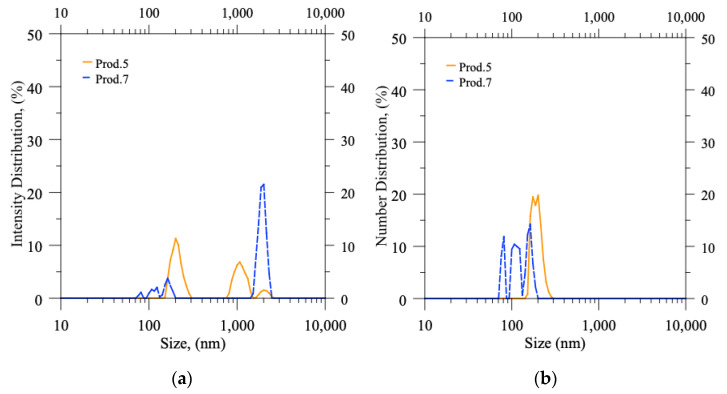
DLS measurements: probability distribution functions (PDFs) in terms of (**a**) Intensity distribution and (**b**) Number distribution versus size of particles suspended in production batches 5–7 (as produced, before filtration).

Production batch 5 was selected as control production due to its best achieved features in terms of e.e. and load, as well as the absence of aggregates. The findings of this investigation (production batch 7) showed an effective load of 0.70% and an e.e. of 29.14% as reported in Table 6. Moreover, the presence of aggregated was detected (see e.s.% value in Table 6 and Figure 9).

This implies that the successful loading of curcumin, as expected, was closely linked to the formation of vesicles too [45]. When the lipid fraction was reduced, there weren’t sufficient bilayers to encapsulate all of the curcumin. As a result, the non-encapsulated CUR began to aggregate upon contact with water, as evidenced by the DLS results presented in Table 7 (and Figure 9). This sequestration, in turn, further impacted the amount of curcumin that could be potentially encapsulated.

The experimental data (values obtained before the syringe filtration step, see Table 5 and Table 7) were subjected to statistical analysis to determine their significance. Specifically, the ratio of CUR/PC (mg of curcumin/mg of phosphatidylcholine) was used as an independent variable, while Z-Average, x_mod,6_, and x_1,0_ were used as dependent variables in three separate series. The statistical analysis was performed using Student’s t-test and one-way analysis of variance (ANOVA), and any differences were considered significant at *p* < 0.05.

The statistical analysis of the data indicated that the results for Z-Average and x_mod,6_ were statistically significant (*p* = 0.005 and *p* = 0.002, respectively, both less than 0.05). This was expected, as the formulative conditions had an impact on the average hydrodynamic size (Z-Average) and the formation of aggregates (related to x_mod,6_).

However, it should be noted that there is always a certain percentage of particles in the small size range (between 100 and 250 nm) regardless of the formulation conditions used. In these cases, there is no statistical significance in the data (as determined by the t-test for the ratio of CUR/PC and x_1,0_ series, with a *p*-value of 0.086, which is greater than 0.05).

**Figure 10:**
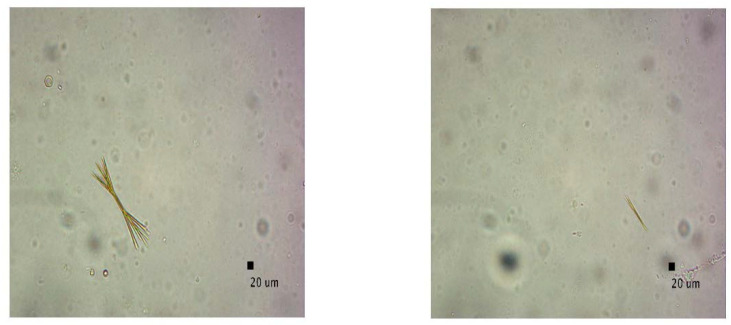
Microscopic pictures of the prod. batch 7 (a dilution of 1:4 and magnification of 40× were applied).

Upon inspections of production batch 7 (as produced, before filtration) by optical microscope, it was observed that the suspensions contained large and small needle-like aggregates (Figure 10), confirming the DLS results. 

The aged products of batch 7 (filtered samples) maintained the same properties of the fresh filtrate, such as size and retention of CUR in vesicles.

## 4. Conclusions

Dosage of curcumin, a powerful active ingredient for pharmaceutical and nutraceutical purposes, is limited by its low bioavailability. Many literature results show that the nanoliposomal curcumin has exhibited great potentiality for several cancer therapies (in in vitro/in vivo tests; clinical trials). Thus, effective, feasible, and sustainable liposomal productions for nanoliposomal curcumin represent a challenge on industrial manufacturing point of view. 

In our research, we have developed and patented a technology called simil-microfluidics, which allows for the rapid and easy production of large quantities of nano-scale liposomal suspensions at room temperature and with minimal energy requirements. Furthermore, the setup simplicity makes the technology an excellent starting point for industrial scalability. In this work, our attention was focused on the production aspects of liposomal curcumin. In particular, by varying curcumin initial concentration, optimized conditions for interesting loads and encapsulation efficiency were defined, and curcumin aggregation formation effect was elucidated. We have found that, by using a precise CUR/lipidic components ratio, nanoliposomes with a load higher than 1% and with a considerable e.e. (roughly 90%) can be obtained. Furthermore, CUR aggregates formation can be avoided, reducing material costs. A productivity of industrial interest, with our apparatus, was confirmed as well as the stability, at refrigerated conditions, of the prepared liposomal suspensions. 

## 5. Patent

Barba A.A., Lamberti G., D’Amore M., Bochicchio S., Dalmoro A., 2018. Process for Preparing Nanoliposomes Comprising Micronutrients and Food Products Comprising Said Nanoliposomes, WO2019049186 (2019)

## Figures and Tables

**Table 1:** Main properties of curcumin [37].

Molecular Formula State	C_21_H_20_O_6_ Solid
Molecular weight (a.m.u.)	368.3799
Water solubility (mg/mL)	insoluble
Solubility in ethanol (mg/mL)	10
Melting Point (°C) Boiling Point (°C)	170–175 (Alfa Aesar, Haverhill, MA, USA) 593.2

**Table 2:** Composition of the seven kind of production batches: weighed Curcumin, PC and Cholesterol and the Effective Concentration (CUR in ethanol considering an initial volume of ethanol of 20 mL).

Batch #	CUR, (mg)	PC, (mg)	CHOL, (mg)	Effective Concentration (CUR in Ethanol) (mg/mL)
Prod. 1	139.8	951.6	176.1	6.99
Prod. 2	99.6	939.7	182.2	4.98
Prod. 3	56.9	934.4	184.7	2.85
Prod. 4	28.3	954.9	187.2	1.42
Prod. 5	14.5	927.4	187.9	0.73
Prod. 6	9.7	945.1	181.6	0.49
Prod. 7	14.2	479.9	95.9	0.71

**Table 3:** Composition of the seven production batches and calculation of the Theoretical Load.

Batch #	CUR, (mg)	PC, (mg)	CHOL, (mg)	Theoretical Load (%)
Unloaded batch	--	950.36	181.5	--
Prod. 1	139.8	951.6	176.1	11.03
Prod. 2	99.6	939.7	182.2	8.15
Prod. 3	56.9	934.4	184.7	4.84
Prod. 4	28.3	954.9	187.2	2.42
Prod. 5	14.5	927.4	187.9	1.28
Prod. 6	9.7	945.1	181.6	0.85
Prod. 7	14.2	479.9	95.9	2.41

**Table 4:** Encapsulation Efficiency and Effective Load of production batches 1–6.

Batch #	e.s. (Syringe)%	e.e. (TFF)%	e.e.%	Theoretical Load%	Effective Load%
Prod. 1	11.13	99.88	11.12	11.03	1.23
Prod. 2	12.97	99.57	12.91	8.15	1.05
Prod. 3	35.51	99.98	35.50	4.84	1.72
Prod. 4	56.34	99.94	56.31	2.42	1.36
Prod. 5	89.61	99.84	89.47	1.28	1.15
Prod. 6	89.74	99.97	89.71	0.85	0.77

**Table 5:** DLS measurements for liposomal suspensions (batches 1 -6) before and after filtration.

Batch #	e.s. (Syringe), %	x_1,0_ (nm)	x_mod,6_ (nm)	Z-Average (nm)	PDI
Cur-liposomes- Prod 1- Before filtration		2159	2154	2348	0.00
Cur-liposomes- Prod 1- After filtration		193	215	315	0.34
Cur-liposomes- Prod 2- Before filtration	12.97	155	1520	705	0.68
Cur-liposomes- Prod 2- After filtration		202	231	227	0.25
Cur-liposomes- Prod 3- Before filtration	35.51	147	1747	889	0.73
Cur-liposomes- Prod 3- After filtration		213	247	224	0.26
Cur-liposomes- Prod 4- Before filtration	56.34	106	1874	1104	0.79
Cur-liposomes- Prod 4- After filtration		226	248	277	0.30
Cur-liposomes- Prod 5- Before filtration	89.61	197	201	351	0.31
Cur-liposomes- Prod 5- After filtration		--	--	345	0.38
Cur-liposomes- Prod 6- Before filtration	89.74	212	231	367	0.39
Cur-liposomes- Prod 6- After filtration		222	215	280	0.30

**Table 6:** Comparison between encapsulation Efficiency and Effective Load of the prod batches 5 and 7.

Batch #	CUR [mg]	PC [mg]	Chol [mg]	e.s. (Syringe) %	e.e. (TFF) %	e.e. %	Theoretical Load %	Effective Load %
Prod. 5	14.5	927.4	187.9	89.61	99.84	89.47	1.28	1.15
Prod. 7	14.2	479.9	95.9	29.15	99.95	29.14	2.41	0.70

**Table 7:** DLS measurements for liposomal suspensions (batches 5–7) before and after filtration.

Batch #	e.s. (Syringe)%	x_1,0_ (nm)	x_mod,6_ (nm)	Z-Average (nm)	PDI
Cur-liposomes- Prod 5 Before filtration	89.61	197	201	351	0.31
Cur-liposomes- Prod 5 After filtration		--	--	345	0.38
Cur-liposomes- Prod 7 Before filtration	29.14	133	2009	897	0.00
Cur-liposomes- Prod 7 After filtration		203	215	340	0.34

## Data Availability

Data sharing not applicable.
